# Supplementary material for: FBXW5 Promotes Tumorigenesis and Metastasis in Gastric Cancer via Activation of the FAK-Src Signaling Pathway
Source: Cancers (Basel). 2019 Jun 17;11(6):836. doi: 10.3390/cancers11060836 (PMC6627937; doi:10.3390/cancers11060836)
Supplement: Supplementary file 1 [file cancers-11-00836-s001.pdf]

Supplementary Materials

FBXW5 Promotes Tumorigenesis and Metastasis in Gastric Cancer via Activation of the FAK-Src Signaling Pathway

Mei Shi Yeo, Vinod Vijay Subhash, Kazuto Suda, Hayri Emrah Balcıoğlu, Siqin Zhou, Win Lwin Thuya, Xin Yi Loh, Sriganesh Jammula, Praveen C. Peethala, Shi Hui Tan, Chen Xie, Foong Ying Wong, Benoit Ladoux, Yoshiaki Ito, Henry Yang, Boon Cher Goh, Lingzhi Wang and Wei Peng Yong

Supplementary Figures and Tables

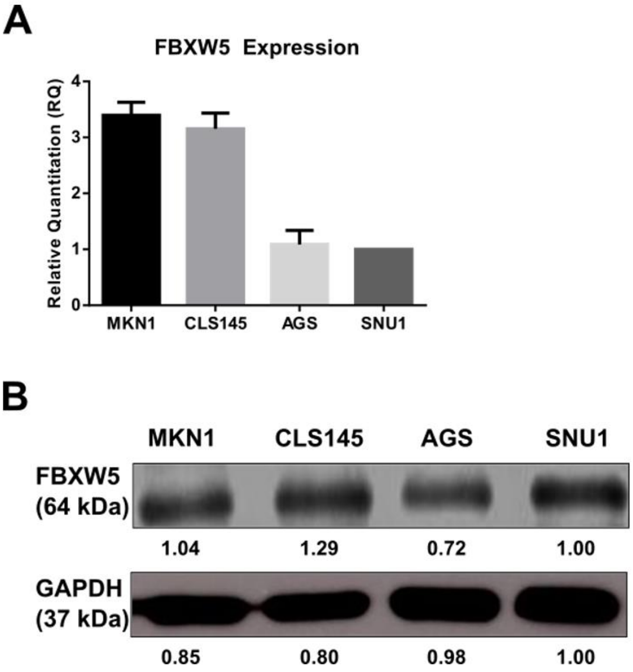

Figure S1. FBXW5 mRNA and protein expressions across gastric cancer cell lines. (**A**) Quantitative real-time PCR analysis of FBXW5 mRNA expression and (**B**) Western blot analysis of FBXW5 protein expression across four gastric cancer cell lines, MKN1, CLS145, AGS and SNU1. Values represent average of three independent experiments and error bars denote standard deviations. GAPDH served as the loading control. At least three independent experiments were performed.

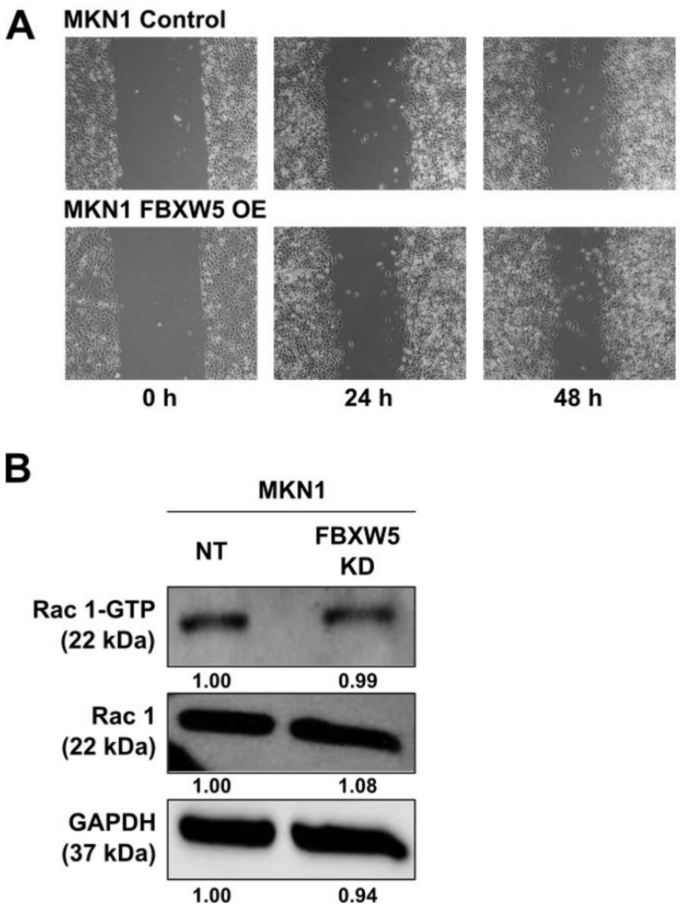

Figure S2. Effect of FBXW5 OE on cell migration and effect of FBXW5 KD on Rac1 activity. (**A**) Phase-contrast microscopic images (10×) of MKN1 control and MKN1 FBXW5 OE cells, captured across three time points after wound was created on the cells. At least two independent experiments were performed. (**B**) Western blot analyses of Rac1–GTP and Rac1 protein expressions in MKN1 NT and MKN1 FBXW5 KD cells. GAPDH served as the loading control. At least two independent experiments were performed.

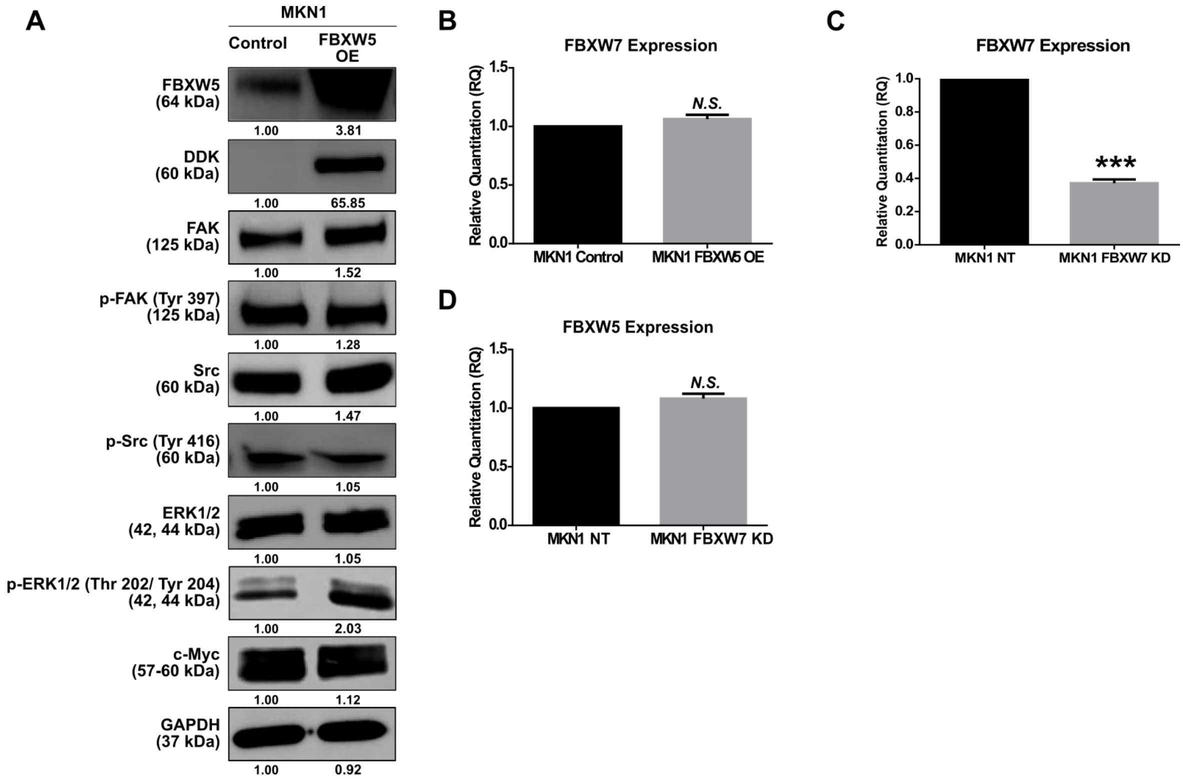

Figure S3. Effect of FBXW5 OE on FAK signaling and FBXW5/ FBXW7 quantitation. (**A**) Western blot analyses of expressions of proteins involved in the FAK signaling pathway in MKN1 control and MKN1 FBXW5 OE cells. GAPDH served as the loading control. At least two independent experiments were performed. (**B**) Quantitative real-time PCR analysis of FBXW7 mRNA expression in MKN1 control and MKN1 FBXW5 OE cells (*p*-value = *N.S.*). Values represent the average of three independent experiments and error bars denote standard deviations. (**C**) Quantitative real-time PCR analysis of FBXW7 mRNA expression in MKN1 NT and MKN1 FBXW7 KD cells (*** *p*-value = 0.0004). (**D**) Quantitative real-time PCR analysis of FBXW5 mRNA expression in MKN1 NT and MKN1 FBXW7 KD cells (*p*-value = *N.S.*). Values represent the average of three independent experiments and error bars denote standard deviations.

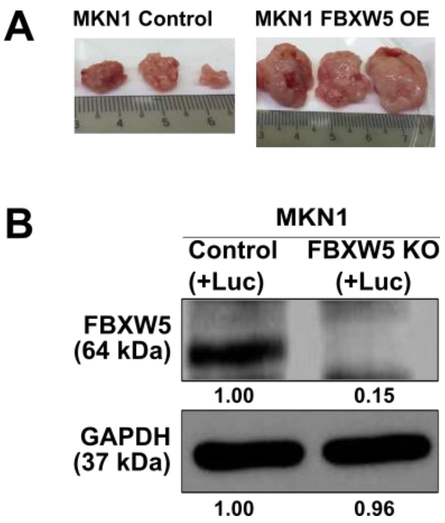

Figure S4. Effect of FBXW5 OE on tumor growth and FBXW5 protein expression in luciferase-tagged MKN1 cells. (**A**) Representative images of tumor of MKN1 control and MKN1 FBXW5 OE cells harvested post subcutaneous implantation into NOD-SCID mouse model. Five animals were randomly assigned to each group and treated for 16 days. (**B**) Western blot analysis of FBXW5 in MKN1 control (+Luc) and MKN1 FBXW5 KO (+Luc) cells. GAPDH served as the loading control. At least three independent experiments were performed.

Table S1. Sequence of primers for real-time PCR.

Sequence of Primers for Real-Time PCR	
**FBXW5**	
Forward	CTGGTACGAGGAGTTCCAGC
Reverse	TGCTCCAGATCTTCACAGTGC
**FBXW7**	
Forward	GACGCCGAATTACATCTGTC
Reverse	GTAGCAGGTCTTTGGGTTC
**GAPDH**	
Forward	ACCACAGTCCATGCCATCAC
Reverse	TCCACCACCCTGTTGCTGTA

Table S2. List of primary and secondary antibodies for Western blot.

Primary Antibodies for Western Blot	
anti-FBXW5<br>anti-Rac1	Thermo Fisher Scientific
anti-FAK (D2R2E)<br>anti-phospho-FAK (Tyr 397)<br>anti-Src (36D10)<br>anti-phospho-Src (Tyr 416)<br>anti-p44/42 MAPK (Erk1/2)<br>anti-phospho-p44/42 MAPK (Erk1/2) (Thr202/Tyr204)<br>anti-c-Myc<br>anti-p21 Waf1/Cip1 (12D1)<br>anti-survivin (71G4B7)<br>anti-phospho- Myosin Light Chain 2 (Ser 19)<br>anti-Cdc42 (11A11)<br>anti-RhoA (67B9)<br>anti-GAPDH	Cell Signaling
anti-DDK	OriGene
anti-ROCK1	Santa Cruz
anti-actin	Cytoskeleton
**Secondary Antibodies for Western Blot**	
anti-rabbit IgG, HRP-Linked<br>anti-mouse IgG, HRP-Linked	Cell Signaling

Supplementary Materials and Methods

1. Wound Healing Assay

Changes in rates of cell migration as a result of FBXW5 overexpression was analysed by a wound healing assay. Cell migration was evaluated by images captured by an inverted fluorescence microscope (Olympus) at 0, 24 and 48 h after wound was created on the cells using the tip of a yellow pipette tip.

2. Small Rho-GTPase Pull-Down Assay

Small Rho GTPase pull-down assays were performed according to the manufacturer's protocol to detect changes in the activities of small Rho-GTPases after FBXW5 knockdown. At 36 h post siRNA transfection, cells were washed with ice-cold TBS and lysed in Lysis/ Binding/ Wash Buffer. Cell lysates were pelleted down by centrifugation and a sample of the cell lysate was used for protein assay using the Pierce BCA Protein Assay. 500 μg of total proteins were incubated with GST-PAK1-PBD (Thermo Fisher Scientific) containing gluthatione resin at 4 °C for 1 h with gentle rocking. Samples were washed four times with Lysis/ Binding/ Wash Buffer. Subsequently, protein expressions of GTP-bound Cdc42 were detected by SDS-PAGE and Western blotting.

3. Preparation of Tissue Samples

Tumor samples harvested were fixed overnight in 4% paraformaldehyde. After overnight fixation in 4% paraformaldehye, tumor samples were then embedded in paraffin and sectioned at 4 μm.

4. Immunohistochemistry (IHC) Staining

Tissue slides were heated at 60 °C for 15 min in order to facilitate tissue attachment and the paraffin softening. Deparaffinization and rehydration were performed with histoclear and in the order of decreasing ethanol concentration. Heat-induced antigen retrieval was done in a microwave oven with DakoCytomation Target Retrieval Solution Citrate pH6 solution (Dako, Denmark) at 120 °C for 15 min to explore antigenic sites. Peroxidase Block Solution (Dako, Denmark) was applied to overlay onto the tissue and incubated in a humidified container at room temperature for 10 min to suppress endogenous peroxidase activity so as to reduce background staining. Sections were then washed twice for 5 min with 1× TBS washing buffer and incubated in a humidified container with monoclonal mouse anti-human Ki67 primary antibody (Dako, Denmark) in the solution of Antibody Diluent with reducing background components (Dako, Denmark) at 4 °C overnight. The sections were again washed twice for 5 min with 1× TBS washing buffer and incubated in a humidified chamber with horseradish peroxidase (HRP) linked anti mouse secondary antibody (Dako, Denmark) at room temperature for 1 h and washed twice with 1× TBS washing buffer, then stained with DAB reagent (Dako, Denmark). Tissue sections were mounted under glass coverslips with DPX (Leica, Germany) after counterstaining with hematoxylin, dehydrating in a series of increasing ethanol concentration and histoclear.

5. Haematoxylin and Eosin (H&E) Immunostaining

Deparaffinization was performed with xylene and in the order of decreasing ethanol concentrations. The tissue sections were then washed twice for 3 min with MiliQ water. Following that, the tissue sections were stained in hematoxylin (Dako), ammonium hydroxide and eosin (Leica) for 1 min each. Subsequently, paraffinization was again performed in the order of increasing ethanol concentrations and xylene. Lastly, tissue sections were mounted under glass coverslips with Eukitt® quick-hardening mounting medium (Sigma Aldrich).

© 2019 by the authors. Licensee MDPI, Basel, Switzerland. This article is an open access article distributed under the terms and conditions of the Creative Commons Attribution (CC BY) license (http://creativecommons.org/licenses/by/4.0/).
